# Barriers to Sustained Implementation of STAR-VA and Strategies to Overcome Them

**DOI:** 10.1093/geroni/igab046.2063

**Published:** 2021-12-17

**Authors:** Jennifer Sullivan, Omonyele Adjognon, Jacquelyn Pendergast, Laura Wray, Michele Karel, Kimberly Curyto

**Affiliations:** 1 VA Providence Medical Center LTSS COIN and Brown University, Providence, Rhode Island, United States; 2 CHOIR, VA Boston Healthcare System, Boston, Massachusetts, United States; 3 CHOIR, VA Boston healthcare System, Boston, Massachusetts, United States; 4 VA Center for Integrated Healthcare, Buffalo, New York, United States; 5 Veterans Health Administration, Washington, District of Columbia, United States; 6 Center for Integrated Healthcare, VA Western NY Healthcare System, Batavia, New York, United States

## Abstract

STAR-VA is an evidence-based, interdisciplinary program helping CLC teams effectively manage DBD. We conducted interviews with 42 key informants involved with STAR-VA implementation in 20 CLCs, guided by a sustainment framework, to understand facilitators and barriers to sustained implementation. We used directed content analysis to identify barriers and mapped them to the CFIR-ERIC Mapping Tool to identify associated implementation strategies. We identified six barriers: 1) staffing issues, 2) lack of written policies, 3) staff buy-in, 4) limited leadership support, 5) exclusion of STAR-VA criteria in performance evaluations, and 6) service line silos. We identified six strategies to overcome these barriers, three strategies most frequently mapped to reported barriers to STAR-VA sustainment: 1) assessing local CLC readiness, facilitators and addressable barriers; 2) identifying and preparing new champions; and 3) altering incentive/allowance structures. The identified strategies can be packaged to further integrate STAR-VA into usual CLC care processes to optimize program sustainability.

